# Convex hulls in hamming space enable efficient search for similarity and clustering of genomic sequences

**DOI:** 10.1186/s12859-020-03811-z

**Published:** 2020-12-30

**Authors:** David S. Campo, Yury Khudyakov

**Affiliations:** Molecular Epidemiology and Bioinformatics, Division of Viral Hepatitis, Centers for Disease Control and Prevention, Atlanta, GA USA

**Keywords:** Population distance, Hamming, Clustering, Centrality

## Abstract

**Background:**

In molecular epidemiology, comparison of intra-host viral variants among infected persons is frequently used for tracing transmissions in human population and detecting viral infection outbreaks. Application of Ultra-Deep Sequencing (UDS) immensely increases the sensitivity of transmission detection but brings considerable computational challenges when comparing all pairs of sequences. We developed a new population comparison method based on convex hulls in hamming space. We applied this method to a large set of UDS samples obtained from unrelated cases infected with hepatitis C virus (HCV) and compared its performance with three previously published methods.

**Results:**

The convex hull in hamming space is a data structure that provides information on: (1) average hamming distance within the set, (2) average hamming distance between two sets; (3) closeness centrality of each sequence; and (4) lower and upper bound of all the pairwise distances among the members of two sets. This filtering strategy rapidly and correctly removes 96.2% of all pairwise HCV sample comparisons, outperforming all previous methods. The convex hull distance (CHD) algorithm showed variable performance depending on sequence heterogeneity of the studied populations in real and simulated datasets, suggesting the possibility of using clustering methods to improve the performance. To address this issue, we developed a new clustering algorithm, *k*-hulls, that reduces heterogeneity of the convex hull. This efficient algorithm is an extension of the k-means algorithm and can be used with any type of categorical data. It is 6.8-times more accurate than *k*-mode, a previously developed clustering algorithm for categorical data.

**Conclusions:**

CHD is a fast and efficient filtering strategy for massively reducing the computational burden of pairwise comparison among large samples of sequences, and thus, aiding the calculation of transmission links among infected individuals using threshold-based methods. In addition, the convex hull efficiently obtains important summary metrics for intra-host viral populations.

## Background

Comparison of large samples of diverse genetic variants and obtaining summary statistics are common tasks in many fields, from computational searches of databases to cancer research and molecular epidemiology. For instance, comparison between populations of mitochondrial DNA variants from normal tissues and tumours brings important insights [1].

Phylogenetic analysis of viral sequences is frequently used in investigation of outbreaks and transmission chains [2–6], usually using a single sequence per infected individual. However, many viruses such as hepatitis C virus (HCV) exist as a population of numerous genetic variants in each infected individual. It was observed that minority variants in the source are often the ones responsible for transmission, indicating that the use of a single sequence per individual lacks sensitivity for detecting such transmissions [7]. Molecular analysis of intra-host viral populations sampled from infected persons was shown to be very efficient in detection of HCV transmissions in outbreak investigations [8–11]. Statistical analysis of intra-host HCV variants obtained from epidemiologically characterized outbreaks allowed for the development of a simple and accurate threshold-based approach for detecting HCV transmissions [7].

Sampling of intra-host HCV variants, including minority variants, by Ultra-Deep Sequencing (UDS) improves sensitivity of transmission detection [7]. However, comparing all sequences from each pair of samples creates a considerable computational challenge. For instance, a relatively small dataset of 401 HCV samples required to perform 80,200 pairwise sample comparisons, for a total of 4.56 × 10^10^ pairwise sequence comparisons [12]. As the number of tested cases increases over time, detection of transmission networks becomes computationally impractical. However, we have observed that less than 1% of all sample-pairs are usually linked by transmission, even in high-risk populations. Therefore, an exhaustive search over all pairs of sequences is very inefficient because the great majority of sample pairs are above certain relatedness threshold, which corresponds to 3.77% for HCV [7]. Briefly, it would be very advantageous to remove most of these pairs in order to reduce the number of computations needed to establish transmission on a set of samples.

Similar problems are encountered under different names in various areas of computer science [13–19]. There are many methods for rapid string comparison. Detection of viral transmissions is affected by three factors: (1) owing to a continuous addition of new data samples during outbreak investigations, there is a need of comparing two or more populations of homologous sequences, which is different from comparison of a sequence to a static data structure or a static database; (2) a need of exact distance calculations makes any available fast heuristic and approximate methods unsuitable for the purpose; and (3) use of the relatedness threshold allows for application of filtering strategies. These factors have been addressed by three previously published filtering algorithms: the *k*-mer bloom filter based on a lower bound of the maximal common substring [20]; the Hamming radius of each population and the triangle inequality [20]; and the signature method based on comparing *k*-mers and *k*-chunks of every pair of sequences [21].

**Fig. 1:**
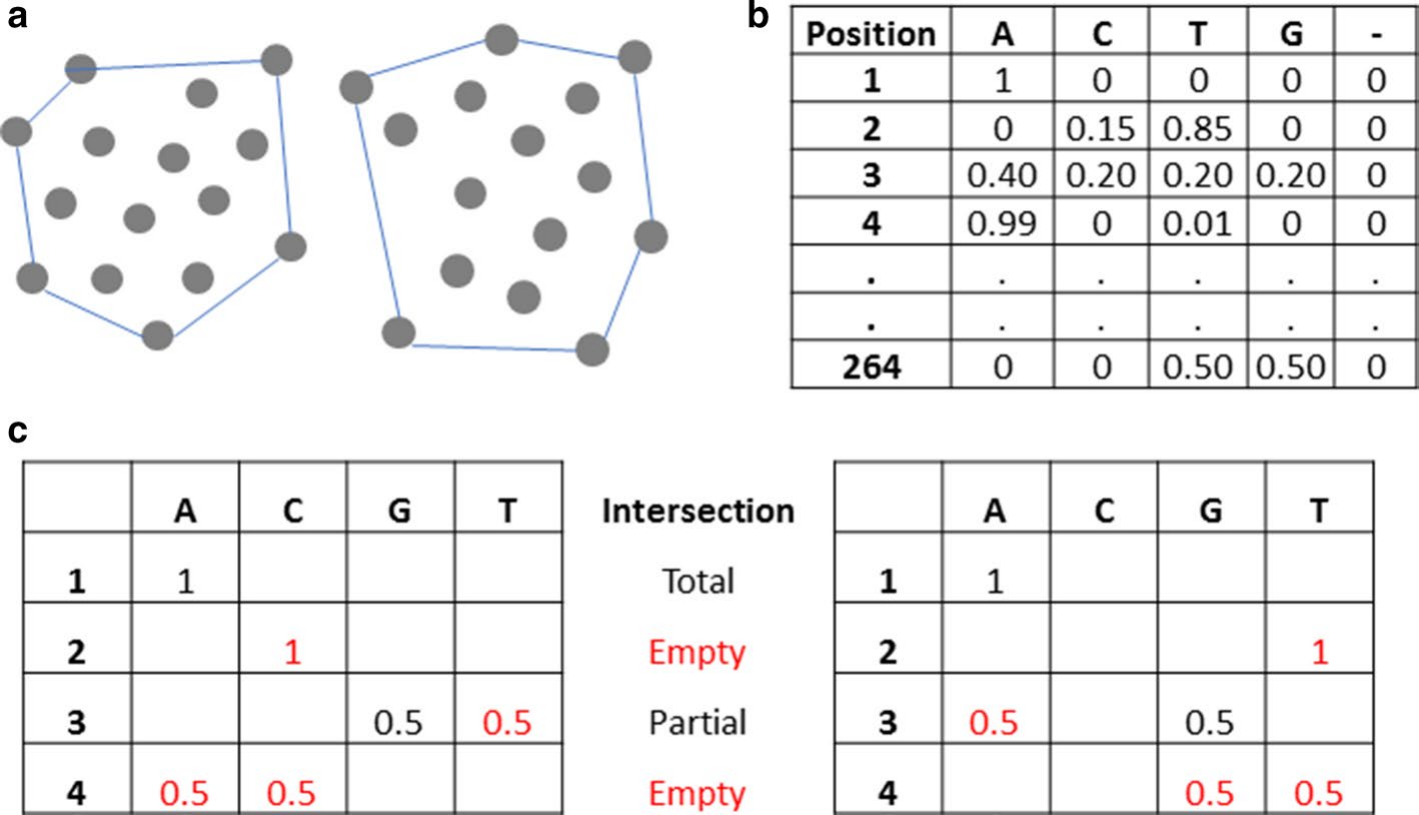
Convex hull data structure. **a** Example of a convex hull in two dimensions. **b** Example of a convex hull frequency vector for a given population. **c** Calculation of the convex hull distance between two populations

The current work was motivated by intuition that comparison of distances among every pair of points in a set can be replaced by calculation of distances among points located at the surface of the set. For the Euclidean plane (Fig. 1a), the convex hull of a set of points X is the smallest convex set that contains X, which can be visualized as the shape enclosed by a rubber band stretched around X [22]. However, every point in the hamming space is “at the surface” [23], which would apparently make this intuition useless for our purposes. Notwithstanding, each convex hull has a schema that describes it succinctly [24]. For instance, for the set of strings Pn = (010000, 011010, 111000, 010010, 011110), the convex hull is *1***0, where * means that the position can be 0 or 1. Thus, the convex hull in the hamming space is a set comprising all binary strings matching the schema (16 sequences in this example).

The Multiple Sequence Alignment (MSA) of nucleotide sequences from viral populations can be used to calculate a vector of frequencies of nucleotide states (A, C, G, T or gap) at each position in the MSA. This is analogous to the convex hull schema described above, with the added information of frequency for each state (Fig. 1b). These vectors are also known as Position weight matrices (PWMs), position-specific scoring matrices or weighted patterns [25]. They have been used in models of transcription factor binding sites and in online matching algorithms [26].

Here, we present a fast and efficient filtering strategy that removes most pairwise comparisons, aiding the calculation of transmission links by threshold-based methods. In addition, we show that application of convex hull significantly reduces the computational cost of many important summary statistics that are routinely calculated for each population.

## Results

### Useful metrics derived from the convex-hull

The convex hull of each file provides the following information:

1. *Average Hamming distance* The average hamming distance (AD_p_) among all sequences within the population *p* is a very common summary statistic of the genetic heterogeneity of a population. However, it is computationally challenging as it requires $\left( {n^{2} - n} \right)/2$ pairwise comparisons and then calculates the average. Using the convex hull, we can obtain AD_p_ in the following manner:
  $$AD{\text{p}} = \frac{{\mathop \sum \nolimits_{a = 1}^{l} \mathop \sum \nolimits_{i \ne j}^{{}} f_{ai} f_{ai} }}{{\left( {n^{2} - n} \right)/2}}$$
  where *n* is the number of sequences, *l* is the length, number of positions, *f*_*ai*_ is the frequency of nucleotide *i* at position *a* and *f*_*aj*_ is the frequency of nucleotide *j* at position *a*.
2. *CC*_*h*_* of each sequence* The average distance from each sequence *h* to all others within the population, its closeness centrality CC_h_, is an important measure of centrality that is also computationally challenging, as we need to make $\left( {n^{2} - n} \right)/2$ pairwise comparisons and then calculate the average of each sequence. Using the convex hull, we can easily obtain CC_h_ in the following manner:
  $$CC_{h} = \mathop \prod \limits_{a = 1}^{l} f_{a}$$
  where *l* is the length (number of positions in the MSA) and *f*_*a*_ is the population frequency of the nucleotide present at sequence *h*, position *a*.
3. *Average distance between two populations* The average hamming distance (AD_pq_) among all sequences of two populations, *p* and *q,* is a very common statistic in population genetics that is the basis for several measures of genetic relatedness. However, it is computationally challenging as it requires $p_{n} *q_{n}$ pairwise comparisons before calculating the average. Using the convex hull, n AD_p_ can be obtained using:
  $$AD{\text{pq}} = \frac{{\mathop \sum \nolimits_{a = 1}^{l} \mathop \sum \nolimits_{i \ne j}^{{}} f_{pai} f_{qai} }}{{n^{2} }}$$
  where *f*_*pai*_ is the frequency of nucleotide *i* at position *a* in the set *p* and *f*_*qaj*_ is the frequency of nucleotide *j* at position *a* in the set *q*.
4. *Lower and upper bound of all distances between two sets* Figure 1c shows the convex hulls of two example populations. In this example, there are 2 positions with empty intersection, therefore, the distance between any sequence in *p* and any sequence in *q* must be 2 *or more*. This is a lower bound of all the distances between members of p and q. This metric helps to safely discard any population pairwise comparison if its value is higher than the threshold T. Thus, the convex hull distance between two populations *p* and *q*, CHD_pq_, can be defined by:
  $$CHD_{pq} = \mathop \sum \limits^{l} a$$
  where *a* is equal to 0 if any nucleotide in position *a* of set *p* is also present in set *q,* or equal to 1 otherwise. If CHD_pq_ > T, this pair of samples can be safely discarded.

In this example there are 3 positions with empty or partial intersection, thus the distance between any sequence in *p* and any sequence in *q* must be 3 *or less*. This is the upper bound of all the distances between members of *p* and *q*.

### Filtering performance

**Fig. 2:**
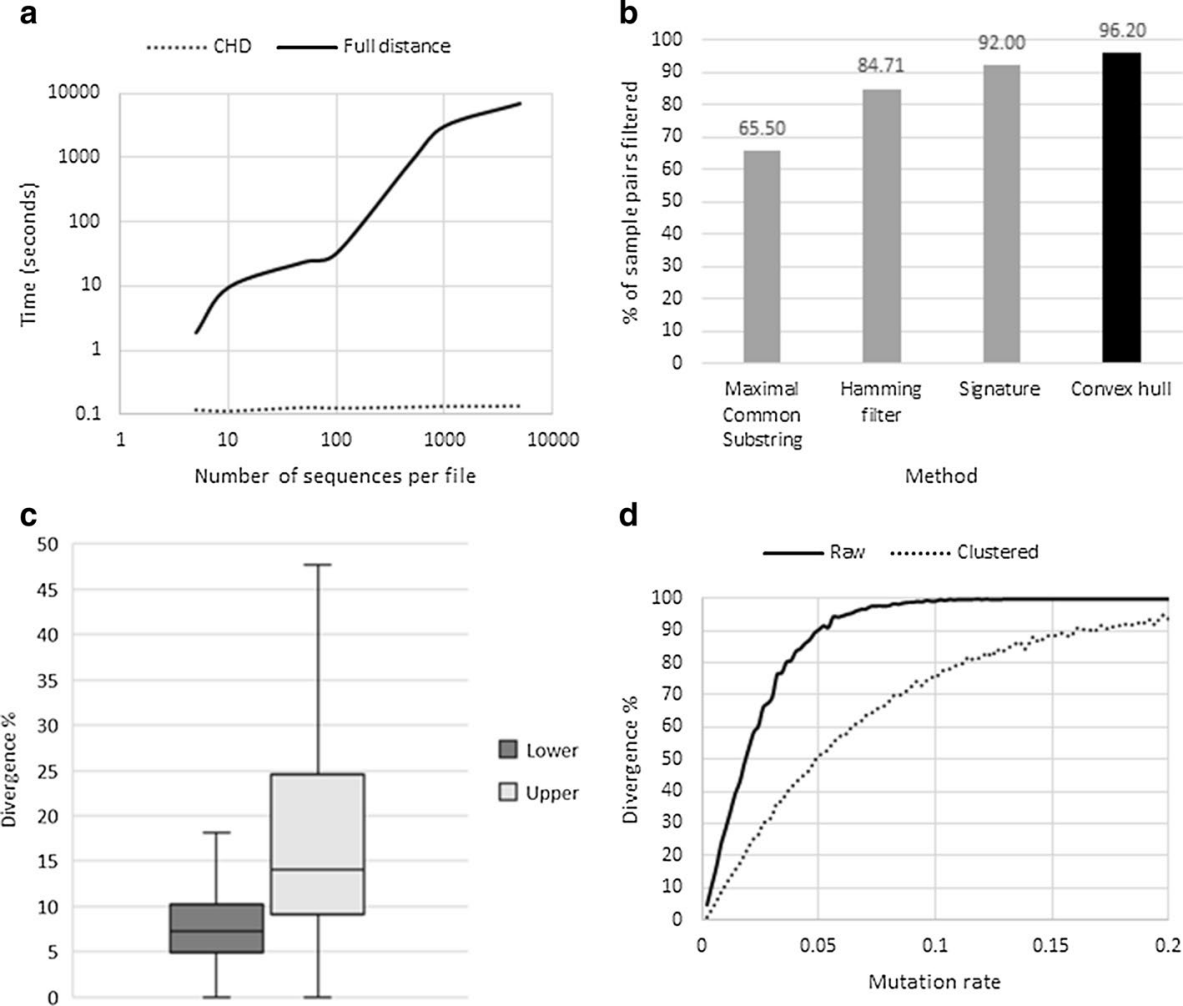
Convex hull distance (CHD) performance. **a** Time taken by the comparison of 20 files with a variable number of sequences. **b** Comparison of filtering performance of four methods. **c** Boxplot of the divergence between the CHD and the actual minimal distance (dark grey) in the viral dataset, as well as the upper bound and the maximal distance (light grey). **d** Divergence percentage between the observed minimal distance and the CHD, for raw samples (continuous line) and samples partitioned into two clusters (dashed line)

To illustrate a greater efficiency of the filtering approach over the full calculation of all sequence pairs, we generated 20 files with a variable number of sequences and applied both algorithms. The time taken by the CHD filter grows very slowly when the number of sequences per file increases, because the number of pairwise sample comparisons remains constant, whereas the full-distance method increases in a quadratic manner (Fig. 2a).

For the viral dataset, the CHD filtering algorithm quickly removed 96.2% of all sample-pairs, outperforming the other three algorithms used here for comparison (Fig. 2b). In the viral dataset, the average divergence between CHD and actual minimal distance was only 7.84% (Fig. 2c), whereas divergence between the upper bound and maximal distance was 2.3-times greater (18.32%).

We performed simulations to test performance of the CHD distance with 100 varying levels of genetic heterogeneity (Fig. 2d). As expected, the greater the diversity, the greater the divergence between true minimal distance and CHD.

### *k*-Hulls performance

**Fig. 3:**
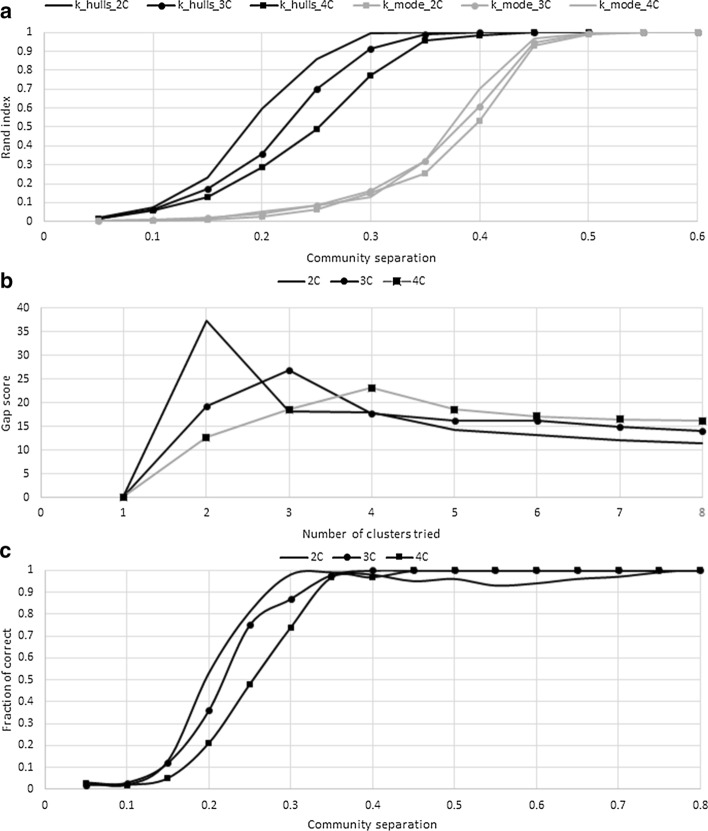
k-hulls performance. **a** Comparison of k-hulls and k-modes for 2, 3 or 4 clusters. **b** Gap score by number of clusters, average of 100 random simulations with a community separation of 0.4. **c** Fraction of samples where the correct number of clusters was chosen

Splitting each population into clusters creates sets of lower diversity than the original, which could provide a boost in CHD performance. Here, we propose a new way to split populations into clusters: a modified version of the *k*-mode algorithm, where the mode (consensus) of the cluster is replaced with the convex hull of the cluster, thus using more information. We performed simulations to test performance of the *k*-hulls algorithm with varying levels of cluster separation (Fig. 3a). Each simulated dataset contained either 2, 3 or 4 true clusters. The *k*-hulls method was found to be consistently correct in classification of sequences into clusters when the community separation was > 0.25. A community separation of 0.25 means that only 25% of the total variation is found between clusters and 75% of the variation is random.

At community separations > 0.5, both the *k*-mode and *k*-hulls approaches were equal in performance. However, for lower values of community separation, *k*-hulls is 6.8-times more accurate. We observed that a higher sensitivity was because *k*-hulls uses more data (all sequences of the cluster instead of just its consensus) and thus avoids many ties in the cluster assignments.

Although algorithms of the k-means family can be performed several times from random starting points, we proposed a heuristic to find the initial seeds that makes use of the closeness centrality calculated using the convex hull. In order to evaluate its performance, we used all possible sets of seeds and ranked them by their average distance. It was found that, in average, our approach identifies a set of seeds outperforming 99.9946% of all other sets.

The main problem of clustering is the difficulty of choosing the optimal number of clusters, as the clusters’ quality always improves (by any intra-cluster or inter-cluster measure) the number of clusters is increased. We used the gap statistic [27] to choose the best clustering solution, in terms of how different it is from a random solution of the same size. Figure 3b shows how the average gap score for 100 random simulations (with a community separation of 0.4) changes with different cluster solutions. For each number of true clusters (n = 2, 3 or 4) the correct solution has the highest gap score. Over all levels of community separation, we found that the method tends to choose the correct number of clusters when the community separation is > 0.35. Regarding the membership within a cluster, we found that geometric mean showed better performance than arithmetic mean due to a reduction in the number of ties.

### *k*-Hulls on HCV dataset

We applied the *k*-hulls algorithm to HCV and simulated datasets to evaluate the CHD filtering performance. On the HCV data, clustering results in correct filtering of 99.92% of sample pairs, which is an improvement over the scenario without clustering (96.2%). On the simulated data with no true clusters, a forced clustering into two groups moderately improves the CHD performance while adding very little computational work (Fig. 1d). With low mutation rates (e.g. < 0.05), clustering improves the CHD performance by 2.4-fold. However, it must be considered that the performance of any filtering strategy would be benefited by splitting the population into clusters, but we only measured its effect on the CHD method.

## Discussion

The convex hull in hamming space is a simple data structure that is efficient in generating information on: (1) average hamming distance within the set, (2) average hamming distance between two sets; (3) closeness centrality of each sequence; and (4) lower and upper bound of all the pairwise distances among the members of two sets. These metrics are routinely calculated by measuring all possible sequence pairs. The approach described here significantly reduces the number of pairs required to be assessed to accurately calculate these metrics. Although we have not provided formal proofs for these shortcuts, all these formulas were tested for correctness by comparing their results with the ones obtained by using the full calculation, with identical results. These comparisons were performed on all datasets, both with the real HCV datasets and simulated datasets produced with a variety of diversity levels.

The CHD filtering algorithm showed the best performance among the studied methods. CHD is also the simplest, which results in a fast processing time (< 1 s on a desktop computer to complete analysis of any dataset used here). In addition, the convex hull of each sample generates a very small file that needs to be calculated only once. Storage of this file requires minimal space, making it readily available for repeat usage when new files are deposited to the database.

The CHD filtering strategy proposed here is applicable in many settings. It can be used for the detection of viral transmissions by a threshold-based method [7] or can be applied to any sets of data with common categorical variables for their rapid comparison.

As expected, a greater diversity results in a greater divergence between the true minimal distance and CHD. Thus, the CHD filtering performance on other viral datasets is expected to be negatively affected by high diversity, for instance, when a patient is infected with > 1 strain. However, this situation can be alleviated by application of clustering algorithms.

Usually, clustering of genomic sequences or other categorical data is performed on distance matrices for all pairs of sequences or variables using methods such as:

1. a similarity tree (e.g. UPGMA) with selection of a level of clustering that satisfies certain constraints (e.g. bootstrap support or a threshold level of difference).
2. a community detection algorithm applied to a network based on the distance matrix.
3. correspondence analysis (like principal component analysis but applied to categorical data using the distance matrix) with the following application of a secondary clustering algorithm.

A different method type, which can be applied to categorical data, is the *k*-mode algorithm [28], which is a version of the *k*-means algorithm applicable to clustering large data sets with categorical values. The algorithm works by calculating a cluster mode of each position. Thus, each sequence is compared to the consensus of a cluster, as opposed to the centroid in continuous data. Here, we improved the *k*-mode algorithm by replacing the consensus with the convex hull of a cluster, thus retaining more information on clusters and achieving a higher accuracy (6.8X) when the community separation is weak. We also showed that the gap statistic provides an efficient way for selection of the best partition solution for the *k*-hulls algorithm.

Before advent of UDS, the detection of viral transmissions was based on phylogenetic analyses of a single viral sequence per patient. Increased UDS sampling of intra-host viral variants from each infected person improved the sensitivity of transmission detection [7] but amplified the computational burden by immensely increasing the number of sequences needed to be compared. Novel molecular surveillance technologies like the Global Hepatitis Outbreak and Surveillance Technology [29] rapidly generate and accrue massive numbers of intra-host viral variants from many infected individuals, presenting significant computational challenges to timely and accurately process molecular data. It is estimated that 2.0–2.8 million people have chronic HCV infection in the United States [30]. Efficient molecular surveillance on such a large population would necessitate fundamental improvements in the capacity to manage and analyze rapidly growing molecular data to assist in devising public health interventions to control and eliminate viral diseases. Computational approaches reducing the computational burden caused by massive data, such as our approach presented here, are important for enhancing surveillance efforts.

## Conclusions

We present a fast and efficient filtering strategy that drastically reduces the amount of computation required for comparison of large samples of genomic sequences. The proposed filtering algorithm has many applications in different fields dealing with comparison of massive datasets of categorical variables such as analysis of sequences of intra-host viral variants for the detection of transmission and transmission networks. In addition, this approach is efficient in calculation of important summary metrics for viral populations.

## Methods

### Problem definition

Given P = (P_1_, P_2_,…), a set of samples where each P_i_ is associated with a set S_i_ = (S_i_^1^, S_i_^2^, …) of homologous sequences, enumerate all sample pairs (P_i_, P_j_) where any pairwise sequence comparisons (S_i_^x^, S_j_^y^) has a hamming distance lower than the relatedness threshold, T (see Fig. 1). Given that every sequence-pair needs to be considered, it yields an O(n^2^) algorithm, where n is the number of sequences.

### Viral dataset

The viral dataset is composed of previously published HCV sequences obtained from 401 HCV infected individuals [7, 12, 20, 21, 31, 32]. The sequences, obtained by UDS, encode the HCV E1/E2 junction (an amplicon of 306 bp, being 264 bp after removing primer sequences), which contains the HCV hypervariable region 1. The average number of unique sequences per sample was 534.3. For each sample-pair, all its sequences are used to create a multiple-sequence alignment (MSA), which is then used to calculate the hamming distance between every pair of sequences. The two samples are considered related if the minimum of the hamming distance between their sequences is smaller than T, which in our case corresponds to 3.77% [7]. All sample-pairs in this viral dataset are above this threshold and thus are unrelated to each other, covering the spectrum of HCV diversity within and between subtypes (1a, 1b, 2a, 3a and 4a).

### Performance comparison

We measure the performance of the method by testing what percentage of all population pairwise comparisons are safely discarded because they cannot have any pair of sequences with a hamming distance below the threshold. The rationale of the approach is that the great majority of sample pairs have sequences that are highly different (unrelated and it would be advantageous to remove these pairs in order to reduce the amount of computation needed to establish transmission on a set of samples. Every sample-pair is still considered, yielding an O(p^2^) algorithm, where p is the number of samples, but a single comparison is made, instead of all sequence pairs. We compare our performance with three previously published algorithms:

1. Maximal common substring [20], based on the lower bound of the maximal common substring, which is applied to the *k*-mer bloom filter.
2. The Hamming radius filter [20], based on the Hamming radius of each population and the triangle inequality.
3. The signature method [21], based on comparing *k*-mers and *k*-chunks of every pair of sequences.

### *k*-Hulls clustering algorithm

The use of a clustering method is expected to improve the performance of the convex hull distance. Here, we modify the *k*-mode algorithm [28] by replacing the consensus cluster with the convex hull of the cluster, thus gaining more information about the cluster. The algorithm is as follows:

1. Choose a desired number of clusters, *k*.
2. Find k seeds:
  a Calculate the convex hull of all data
  b *1st seed* Calculate the closeness centrality of each sequence with the formula described for closeness centrality (CC_h_). The most distant sequence is the 1st seed.
  c *2nd seed* Calculate the distance from the 1st seed to all others and identify the most distant.
  d For each additional seed: Calculate the distance from the previous seeds to all other sequences and find the sequence with the highest geometric mean.
3. Preparations:
  a Transform each sequence into the convex hull format.
  b Each cluster begins with just one seed.
4. While the cluster membership changes:
  a Calculate the Euclidean distance between each sequence’s convex hull and each cluster’s convex hull.
  b Assign the sequence to its closest cluster and obtain a list of cluster memberships.
  c Recalculate the convex hull of each cluster.

In order to find the optimal number of clusters, we used the gap score [27] in the following manner:

1. Calculate the goodness of the clustering solution. For each position on each cluster, calculate the Shannon entropy, then average over all positions.
2. Calculate an average of “within cluster” goodness (rather than “between cluster”).
3. Compare this value with the one obtained by 1000 random partitions of the same size by means of the gap score.
4. Choose the solution with the best score.

### Simulated datasets

To test the performance of the CHD and the k-hulls algorithm, we created several simulated datasets with varying degrees of sequence heterogeneity and variable number of true clusters. For measuring the effect of genetic heterogeneity on the CHD distance, 5000 simulated pairs of files were created, with 50 replicas for each level of mutation rate (100 levels) and with each pair comparing two populations with the same level of simulated diversity.

For the clustering algorithm, 39,900 simulated datasets were created, with 100 replicas for each parameter combination. Each file consisted of 48 sequences, each sequence 50 nucleotides long. The sequences could be separated into 2, 3 or 4 true clusters, with 20 levels of community separation, ranging from 0.05 to 0.95. For instance, a community separation of 0.05 means that only 5% of the total variation is found between clusters and 95% of the variation is random.

## Data Availability

Data and software are available upon request.

## Abbreviations

HCV: Hepatitis C virus
UDS: Ultra-Deep Sequencing
CHD: Convex hull distance
CC: Closeness centrality
PWM: Position weight matrices
MSA: Multiple Sequence Alignment
AD: Average distance
